# Experimental demonstration of spatiotemporal analog computation in ultrafast optics

**DOI:** 10.1038/s41377-025-02109-0

**Published:** 2026-01-22

**Authors:** Junyi Huang, Dong Zhao, Jixuan Shi, Hongliang Zhang, Hengyi Wang, Fang-Wen Sun, Qiwen Zhan, Shiyao Zhu, Kun Huang, Zhichao Ruan

**Affiliations:** 1 School of Physics, State Key Laboratory of Extreme Photonics and Instrumentation, and Zhejiang Province Key Laboratory of Quantum Technology and Device, Zhejiang University, Hangzhou, 310027 China; 2 Department of Optics and Optical Engineering, University of Science and Technology of China, Hefei, 230026 China; 3 Department of Physics, Tsinghua University, Beijing, 10010 China; 4 Hefei National Laboratory, Hefei, China; 5 School of Optical-Electrical and Computer Engineering, University of Shanghai for Science and Technology, Shanghai, 200093 China; 6 Zhejiang Key Laboratory of 3D Micro/Nano Fabrication and Characterization, Department of Electronic and Information Engineering, School of Engineering, Westlake University, Hangzhou, 310027 China; 7 College of Optical Science and Engineering, Zhejiang University, Hangzhou, 310027 China; 8 State Key Laboratory of Opto-Electronic Information Acquisition and Protection Technology, School of Physical Sciences, University of Science and Technology of China, Hefei, Anhui 230026 China

**Keywords:** Applied optics, Imaging and sensing, Nanophotonics and plasmonics

## Abstract

It is intractable to perform information processing and computation on single ultrafast optical pulses, within picoseconds or even femtoseconds. Here, we experimentally demonstrate an optical spatiotemporal differentiator, a mirror-symmetry-breaking dielectric metagrating, which performs analog computations of both spatial and temporal differentiations on single ultrafast optical wavepackets. The spatiotemporal differentiator is designed with a transfer function with linear dependence on spatial wavevector and temporal frequency and fabricated by using a double-exposure E-beam lithography process. We achieve the first-order spatiotemporal differentiation with experimental resolutions of approximately 14 μm (in space) and 260 fs (in time). Furthermore, we report a parabolic relationship between the transverse velocity of a front-tilted photonic wavepacket and the normalized intensity of its first-order spatiotemporal-differentiation wavepacket. This relationship allows direct measurement of the transverse velocity using only the normalized intensity, fundamentally simplifying velocity detection. These capabilities of optical spatiotemporal computation endow emerging space-time optics with fundamental computation blocks.

## Introduction

Spatiotemporal structured light can provide new capabilities in controlling ultrafast optical pulses as required^1–7^. Several studies have addressed the great opportunities for joint manipulation on both spatial and temporal degrees of freedom, where the space-time coupling effect enables novel phenomena, including optical space-time wavepackets^8–11^, front-tilted pulses^12,13^, full spatiotemporal light modulation^14,15^, spatiotemporal optical vortices (STOVs)^16–22^, and so on. More recently, spatiotemporal holograms^14,23,24^, spatiotemporal images^25–27^, and STOV arrays^14,25^ have been encoded in a single ultrafast optical pulse, with critical potential applications in optical communication, high-resolution microscopy, high-harmonic generation, and laser micromachining.

Since spatiotemporal optical wavepackets are just within the time scale of picoseconds or even femtoseconds, high-speed information processing to these ultrafast optical pulses is far beyond the capability of state-of-the-art electronics. Recently, substantial interest has been sparked to explore nanophotonic structures with analog computation features, which outperform conventional digital processing to optical fields^28,29^. Extensive studies show that optical differentiators enable parallel image processing to extract the boundary of objects, and achieve high computation efficiency without analog-to-digital conversion^30–38^. In particular, special designed structures have been proposed to implement analog computation to spatiotemporal wavepackets^39–43^. For example, with a mirror-symmetry-breaking metagrating, we proposed the nonlocal effect to enable optical spatiotemporal differentiation, which could be deployed to highlight the space-time variations of ultrafast optical pulses and generate the STOVs^40^. By performing optical computation of high-order spatiotemporal differentiation, a very recent theoretical work discussed event-based image processing metasurfaces when the input image is evolving in time on subpicosecond timescales^44^. A metallic meta-device was fabricated and used to demonstrate spatiotemporal differentiation for microwave signals^43^. For spatiotemporal computation with intractable ultrafast pulses, none of these structures, however, has been demonstrated experimentally either.

Here, we experimentally demonstrate an optical spatiotemporal differentiator, a mirror-symmetry-breaking dielectric metagrating, which performs analog computations of both spatial and temporal differentiations to ultrafast optical wavepackets. The spatiotemporal differentiator is designed with a transfer function with linear dependence on spatial wavevector and temporal frequency, and fabricated by using a double-exposure E-beam lithography process. Generally, when a spatiotemporal wavepacket transmits through the metagrating, the output wavepacket exhibits an envelope proportional to the joint-differentiation profile of the incident pulse in both space and time. As an example, we generate front-tilted photonic wavepackets as the incident pulses and measure the spatiotemporal profile of the transmitted ones. Due to the presence of an out-of-phase delay between the spatial and temporal contributions, the metagrating creates spatiotemporal phase singularities with transverse orbital angular momentum of light. Furthermore, we report a parabolic relationship between the transverse velocity of a front-tilted photonic wavepacket and the normalized intensity of its first-order spatiotemporal-differentiation wavepacket. This relationship allows direct measurement of the transverse velocity using only the normalized intensity, fundamentally simplifying velocity detection. Our experimental results demonstrate that compact nanostructures provide fundamental computation blocks for extreme spatiotemporal manipulation, paving the way towards integrated ultrafast optical computing.

## Results

### Design and experimental characterization of spatiotemporal joint manipulation

**Fig. 1:**
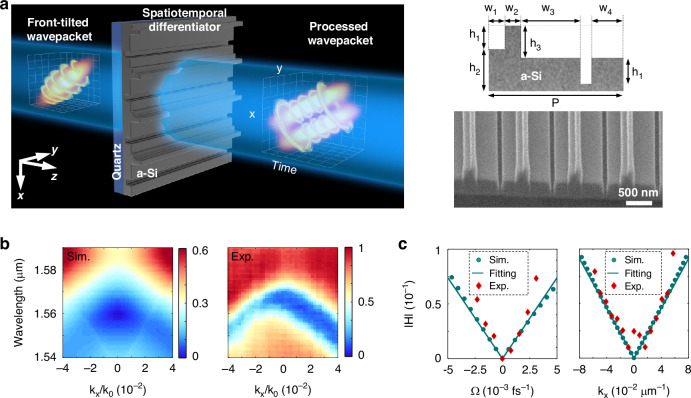
Optical spatiotemporal differentiator for a single ultrafast wavepacket. **a** Designed metagrating computing the first-order spatiotemporal differentiation to an incident wavepacket, where the envelope of the transmitted wavepacket is given as ${S}_{{tran}}\left(x,t\right)=-i{C}_{x}\frac{\partial {S}_{{in}}}{\partial x}+{{iC}}_{t}\frac{\partial {S}_{{in}}}{\partial t}$ for any input wavepacket with the envelope$\,{S}_{{in}}\left(x,t\right)$. Here, we use incident front-tilted photonic wavepackets as a representative example to demonstrate that the spatiotemporal differentiator performs analog computations of both spatial and temporal differentiation on single ultrafast optical wavepackets. Cross section of the unit cell and the SEM image. **b** Simulated and measured amplitude of the transfer function $H$ at different wavelengths and spatial frequencies. **c** A comparison between the simulated and experimental amplitudes of the transfer function along *k*_*x*_ = 0 and *Ω* = 0

To demonstrate the first-order spatiotemporal differentiator, we design a metagrating with silicon nanostructures sitting on a quartz substrate (Fig. 1a). Based on the mirror-symmetry breaking^40^, a spatiotemporal differentiation has a transfer function in frequency space $H={C}_{x}{k}_{x}+{C}_{t}\Omega$, where ${k}_{x}$ is the transverse wavevector, $\Omega =\omega -{\omega }_{0}$ denotes the sideband frequency with the center frequency ${\omega }_{0}$ of the wavepacket, and ${C}_{x}$ and ${C}_{t}$ are the differentiation coefficients in space and time, respectively. As a result, for any input pulse with the envelope$\,{S}_{{in}}\left(x,t\right)$, the envelope of the transmitted pulse is given as ${S}_{{tran}}\left(x,t\right)=-i{C}_{x}\frac{\partial {S}_{{in}}}{\partial x}+{{iC}}_{t}\frac{\partial {S}_{{in}}}{\partial t}$.

To evaluate the space-time coupling, we define a complex parameter $A={C}_{t}/{C}_{x}$. The magnitude of ${|A|}$ is related to the coupling strength, while space-time decoupling occurs if $\left|A\right|=0$ or *∞*. Note that the phase $\Delta \varphi =\arg (A)$ determines the phase delay between the spatial and temporal contributions. By breaking the mirror symmetry of the metagrating, it has been shown that the contribution can be out of phase, i.e., $\Delta \varphi \ne 0$ or $\pi$ (ref. ^40^). For the out-of-phase case, the transfer function $H$ has phase singularity and zero value at ${k}_{x}=0$ and $\Omega =0$. Thus, for a normal incident Gaussian-shaped pulse, the pulse transmitted through the metagrating will be a STOV with the phase singularity (where the zero-value intensity is located) in the spatiotemporal domain, as shown experimentally later.

By using the finite-element method, we design the metagrating on a quartz substrate, with the center operation wavelength at ${\lambda }_{0}=1560$ nm (see the design details in Materials and methods (MM) Sec. 1 and Supplementary Material (SM) Fig. S1). We fabricated experimentally with double-exposure E-beam lithography (EBL) by using two masks (MM Sec. 2). The metagrating with a subwavelength period of $p=1005$ nm diffracts the normally incident light into only the zeroth order due to $p < {\lambda }_{0}$. For breaking mirror symmetry, the unit cell consists of different widths and heights for the gaps and pillars, where the geometric parameters are ${w}_{1}=128$ nm, ${w}_{2}=112$ nm, ${w}_{3}=429$ nm, ${w}_{4}=240$ nm, ${h}_{1}=235$ nm, ${h}_{2}=375$ nm, ${h}_{3}=320$ nm (Fig. 1a).

To measure its transfer function, we develop a homemade angle-resolved optical spectroscopy (MM Sec. 1). Figure 1b shows the transfer function $H$ by simulation and experimental results. Approximately at a central operation wavelength of ${\lambda }_{0}=1560$ nm and ${k}_{x}=0$, the linear dependence of $H$ on the time and spatial frequencies reveals the required feature for a spatiotemporal differentiator (Fig. 1c), and the expected phase singularity is located. To evaluate the experimental ${C}_{x}$ and ${C}_{t}$, both the measured and simulated transfer functions are compared at the temporal and spatial frequencies, where their good agreement confirms the required linear dependence in a first-order spatiotemporal differentiator. We fit the amplitude and phase of $H$ along the lines ${k}_{x}=0$ and$\,\Omega =0$, respectively. This yields theoretical values of ${C}_{x}=1.14{e}^{-1.49i}$ μm and ${C}_{t}=14.8{e}^{-3.06i}$ fs, implying a nonzero space‒time coupling strength of $A\approx -12.98i$ fs μm^−1^. The phase delay between ${C}_{x}$ and ${C}_{t}$ is close to $\pi /2$, which indicates the out-of-phase contribution between the spatial and temporal differentiation computations.

### Spatiotemporal differentiation and phase-singularity generation

**Fig. 2:**
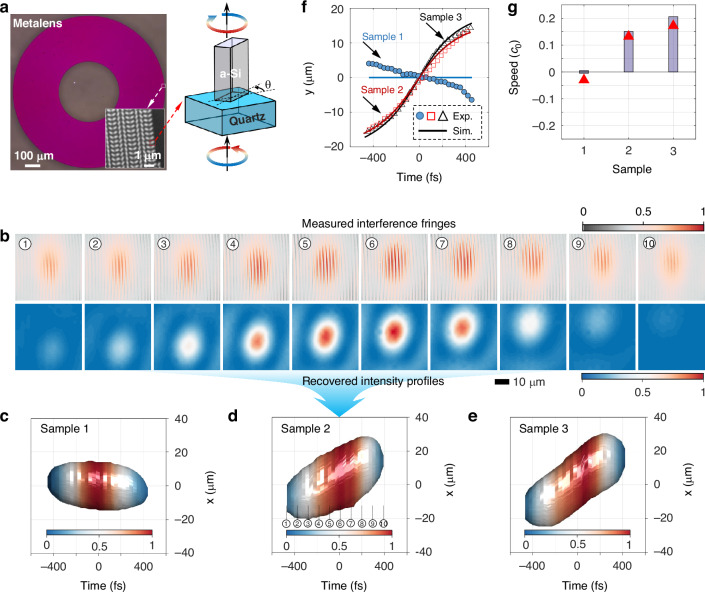
Generation of incident front-tilted wavepackets by using metalens. **a** Microscopy images of our fabricated metalens for generating front-tilted wavepackets. The bottom-right inset shows a zoomed-in microscopy image of the metalens with the geometric phase used in the metalenses. The amorphous silicon film with a thickness of 760 nm is located on a sapphire substrate. Such a Si-nanobrick can work as a miniaturized half-waveplate, which can change the incident circular polarization into cross polarization with an additional phase modulation of twice the rotation angle *θ*. **b** Measured interference fringes (upper panel) and the relative retrieved intensity profiles (lower panel) of the front-tilted wavepackets generated by the metalens in Sample 2. The labels denote the time sequence, which can be addressed in (**d**). The pseudo-colors show the normalized intensity of measured fringes (upper colorbar) and recovered profile (lower colorbar). Retrieved iso-intensity profiles of incident wavepackets generated by different dispersion-engineered metalenses: Sample 1 (**c**), Sample 2 (**d**) and Sample 3 (**e**). The pseudo-colors show the normalized intensity for three cases. **f** Time-dependent centroids of the measured intensities. **g** Transverse speed for three samples for design (bar) and measurement (triangle)

To show the first-order spatiotemporal differentiator performing analog computation, we generate three different front-tilted photonic wavepackets by designing and fabricating high numerical aperture metalenses with off-centered phase profiles (see the design and fabrication details in MM Sec. 2). By controlling the propagation delay, the incident wavepacket at the radial position of the metalenses arrives at the focal plane in different sequential times so that the eccentric phase with *x*-coordinate dependence leads to a laterally moving focus with time, i.e., spatiotemporal front-tilted wavepackets. With different geometric phase distributions (chosen for their dispersion-less feature^45,46^), three samples are designed and fabricated (see Sample 2 in Fig. 2a, and Samples 1 and 3 in SM Fig. S6).

The generated front-tilted wavepackets are characterized with a custom-made Mach–Zender interferometer (SM Fig. S7). By using a delay line to control the signal arm, the upper panel in Fig. 2b shows the measured fringes shift vertically for Sample 2, implying the transverse motion of the wavepackets (see the retrieved intensity profiles in the lower panel in Fig. 2b). The measured results for Samples 1 and 3 are shown in SM Fig. S8, 9, respectively. For a better observation of these transverse shifts, we show the iso-intensity profiles of these retrieved wavepackets with different transverse velocities in Fig. 2c, d, which reveal the expected shift of the intensity profiles with time. The time-dependent centroids of the measured intensities for these wavepackets are in good agreement with their theoretical values (Fig. 2f). By fitting these shifted centroids, we obtain the measured transverse velocities: ${v}_{1}=-0.028{c}_{0}$ (Sample 1), ${v}_{2}=0.1318{c}_{0}$ (Sample 2) and ${v}_{3}=0.1752{c}_{0}$ (Sample 3), as observed in Fig. 2g, where ${c}_{0}$ is the speed of light in vacuum.

**Fig. 3:**
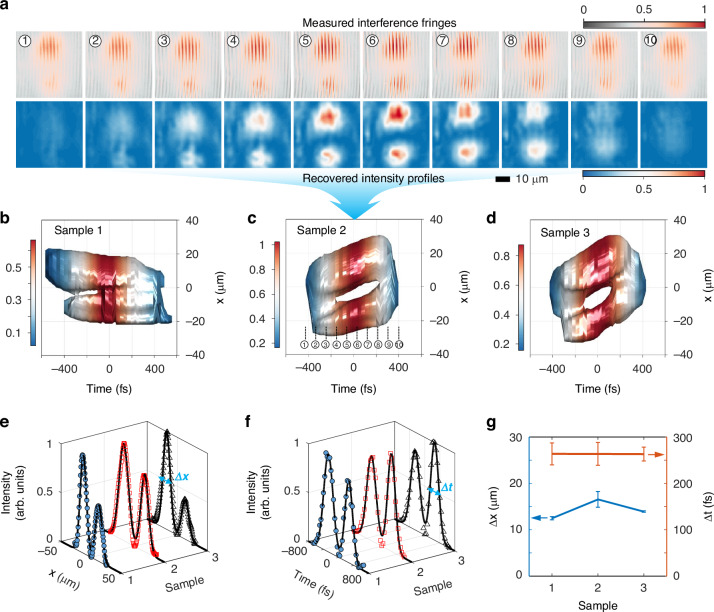
Experimental spatiotemporal differentiation by metagrating. **a** Measured interference fringes (upper panel) and the relative retrieved intensity profiles (lower panel) of the transmitted wavepackets for the incident wavepacket generated by Sample 2. The labels denote the time sequence, which can be addressed in (**c**). The pseudo-colors show the normalized intensity of measured fringes (upper colorbar) and recovered profile (lower colorbar). **b**–**d** Retrieved iso-intensity profiles of the transmitted wavepackets, when the incident wavepackets in Fig. 2c–e passing through our metagrating, respectively. The intensity profiles of these retrieved wavepackets are normalized to the peak intensity of their individual incident wavepackets. The pseudo-colors show the normalized intensity for three cases. Measured intensity profiles along the spatial (**e**) and temporal (**f**) directions. Both intensity profiles are fitted by using a weighted superposition of Gaussian functions (solid black curves). The full width at half maximum (FWHM) of each peak can be used to evaluate the spatial (Δ*x*) and temporal (Δ*t*) resolutions of the differentiations. **g** Measured spatial (left) and temporal (right) accuracies of the differentiations for the different samples. These data are obtained directly from the intensity profiles in (**e**, **f**)

By inserting the metagrating into the signal arm of the Mach–Zehnder interferometer, we observe that the spatiotemporal wavepackets differentiated by the metagrating have interference fringes with two-lobe shapes. The upper panel in Fig. 3a shows the interference fringes when the incident wavepacket is generated by Sample 2 (see SM Fig. S8, 9 for the cases of Sample 1 and 3, respectively). The two lobes in the intensity profile of the transmitted wavepackets are key signatures of spatiotemporal differentiation applied to the incident front-tilted wavepackets, as a result of the differentiation computation performed on the envelope of the input wavepacket. Owing to the transverse velocities of the incident front-tilted wavepackets, the retrieved intensity profiles (the lower panel in Fig. 3a in the case of Sample 2) of the spatiotemporal differentiation wavepackets also shift with time. For a better observation, Fig. 3b–d show the iso-intensity profiles of the transmitted pulse for the incident wavepackets generated by all the three samples, respectively. As one of the features of spatiotemporal differentiation, the zero-intensity profiles in the spatiotemporal-differentiation wavepackets are observed experimentally near the spatial and temporal centers (i.e., *x* = 0 and *t* = 0), where the incident wavepackets have the slowest spatiotemporal variation.

To elaborate such a differentiation operation, the measured *x*-directional (Fig. 3e) and temporal (Fig. 3f) intensity profiles across the zero-intensity position exhibit the expected saddle shapes with two peaks. The full width at half maximum (FWHM) of each peak is used to evaluate the resolution of both spatial and temporal differentiations. By using numerical fitting (see the solid black curves in Fig. 3e, f), we obtain the experimental resolutions Δ*x* and Δ*t* (Fig. 3g) for different samples. The average values are Δ*t* = 263.4 fs and Δ*x* = 14.3 μm, which coincide with the theoretical predictions Δ*t* = 200 fs and Δ*x* = 12.5 μm, derived from the linear-dependence ranges in the temporal frequency and wavevector domains, approximately about $-0.005\le \Omega /{{\rm{\omega }}}_{0}\le 0.005$ and ${-0.08\le k}_{x}/{k}_{0}\le 0.08$ (Fig. 1c). The experimental temporal resolution Δ*t* is greater than the predicted Δ*t*, which is caused by the temporal width of the reference probe in the interferometer. Note that the differentiation resolutions can be further enhanced by designing metagratings with the low Q-factor, which broadens the linear ranges in both the temporal and spatial frequency domains, according to spatiotemporal coupled-mode theory^47–49^.

**Fig. 4:**
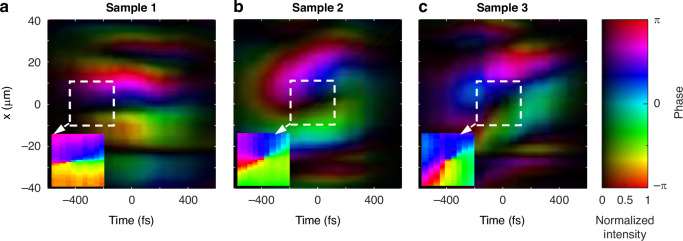
Spatiotemporal phase singularities of the transimtted wavepackets. **a**–**c** Retrieved phase and intensity profiles of the transmitted wavepackets of Fig. 3b–d when the incident wavepackets generated by different samples passing through our metagrating. For a better observation of the spatiotemporal phase singularities, these insets show pure phase profiles in the white-dashed squares

Such a spatiotemporal differentiation exhibits the advantage of optical analog computing, which can process with a few hundred femtoseconds without optical-to-electric and electric-to-optical signal conversion. As another feature of spatiotemporal differentiation, spatiotemporal phase singularities are also revealed experimentally by using the retrieved phase and intensity profiles (Fig. 4). Near the centers, we can observe the phase singularities with dark intensity profiles of the transmitted wavepackets, which originate fundamentally from the out-of-phase delay between the spatial and temporal differentiation. Correspondingly, across these phase singularities, the measured interference fringes have half-period dislocations between the upper and lower parts of the fringes, implying an expected phase jump of π over the singularities. These observations agree with the null intensities around the centers as depicted in the iso-intensity contours (Fig. 3b–d). Thus, we have confirmed the validity of the proposed spatiotemporal differentiator to the incident front-tilted wavepackets. For general spatiotemporal wavepackets, the spatiotemporal computation can be verified by measuring the profiles of both the incident and transmitted pulses using an experimental setup similar to the Mach–Zehnder interferometer shown in SM Fig. S7.

### Probing ultrafast transverse shifting velocity by spatiotemporal differentiation

Given that practical laser wavepackets have near-Gaussian spectra^50^, we consider a front-tilted photonic wavepacket with a Gaussian-type temporal distribution and a general transverse envelope transversely shifting

1 $${S}_{{in}}\left(x,{t;}{v}_{T}\right)=f\left(x{-v}_{T}(t-{t}_{0})\right)\cdot {e}^{-{\left(\frac{t-{t}_{0}}{{w}_{t}}\right)}^{2}}$$

where $x$ is only the lateral coordinate to simplify the discussion, ${v}_{T}$ is the transverse shifting velocity of the front-tilted wavepacket, and ${w}_{T}$ and ${t}_{0}$ denotes the waist radius and the center of the Gaussian distribution in the temporal dimension, respectively. The general function $f(x)$ is the spatial distribution of the wavepacket at the time $t={t}_{0}$. Note that ${v}_{T}$ determines only the transverse velocity of the pulse with respect to the time axis, which reflects the inherent shape of the pulse. Therefore, the velocity ${v}_{T}$ is independent of the propagation velocity of the pulse. The transmitted wavepacket is

2 $${S}_{{tran}}\left(x,{t;}{v}_{T}\right)={-{iC}}_{x}{e}^{-{\left(\frac{t-{t}_{0}}{{w}_{t}}\right)}^{2}}\cdot \left[{\left(1+A{v}_{T}\right)\cdot f}^{{\prime} }\left(\xi \right)+\frac{2A(t-{t}_{0})}{{{w}_{t}}^{2}}f\left(\xi \right)\right]$$

where ${f}^{{\prime} }$ is the first-order differentiation of the function $f$, $\xi =x{-v}_{T}(t-{t}_{0})$ and the coupling coefficient $A={C}_{t}/{C}_{x}$. The magnitude $\left|A\right|$ is related to the coupling strength between space and time. Equation (2) expresses the dependence of the transmitted wavepacket on the transverse velocity ${v}_{T}$. To obtain a measurable parameter, the normalized intensity is given as

3 $$\bar{I}\left({v}_{T}\right)=\frac{I({x,t}_{0};{v}_{T})}{I({x,t}_{0};0)}={\left|1+A{v}_{T}\right|}^{2}$$

where the intensity of the transmitted wavepacket is $I\left(x,{t;}{v}_{T}\right)={\left|{S}_{{tran}}\left(x,{t;}{v}_{T}\right)\right|}^{2}$. We note that at time $t={t}_{0}$, the first-order derivative of the intensity for the transmitted wavepacket is zero (see the details in SM Sec. 2), which means a maxima or minima of intensity, and this property can be utilized to determine the time ${t}_{0}$.

Note that Eq. (3) indicates that the transverse velocity can be inferred by measuring the intensity at one moment, without the requirement for measurements of their whole temporal and spatial profiles. First, since the suggested $\bar{I}\left({v}_{T}\right)$ is independent of the spatial coordinates *x*, we can use the integrated intensity $P\left({t;}{v}_{T}\right)=\int I(x,{t;}{v}_{T}){dx}$ in experiment, leaving one-dimensional temporal intensity and have that$\,\bar{I}\left({v}_{T}\right)=P({t}_{0};{v}_{T})/P({t}_{0};0)={|1+A{v}_{T}|}^{2}$. Such an operation facilitates experimental implementation because it can suppress the errors of intensity from spatial deviation and inevitable environmental noise. Second, $\bar{I}\left({v}_{T}\right)$ depends on only the transverse velocity ${v}_{T}$ when the coupling coefficient $A$ is fixed for a given spatiotemporal differentiator. Once the dimensionless parameter $\bar{I}\left({v}_{T}\right)$ is obtained by measuring the integrated intensity $P$ at time $t={t}_{0}$, we can calculate the transverse velocity ${v}_{T}$ directly via Eq. (3). Third, Eq. (3) shows that the coupling between spatial and temporal differentiations is significant for the proposed mapping, because when they are decoupled, i.e.,$\left|A\right|=0$ or ∞, it will be invalid to measure ${v}_{T}$.

**Fig. 5:**
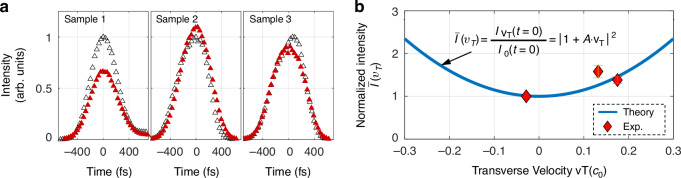
Measuring transverse shifting speed via spatiotemporal differentiations. **a** Measured time-dependent intensity profiles of the incident (hollow triangles) and differentiated (solid red triangles) wavepackets after integration with respect to the spatial coordinates. **b** Measured (diamonds) and predicted (curves) parabolic relationships between the normalized peak (which is located at $t=0$ in (**a**) intensity and transverse shifting speed

To demonstrate this effect, we verify the parabolic relationship in Eq. (3) by measuring the intensity values when the front-tilted wavepackets transmit through the spatiotemporal differentiator, with different transverse velocities. To eliminate the deviation from different powers in each incident laser wavepacket, the experimental $P\left({t;}{v}_{T}\right)$ (solid red triangles in Fig. 5a) of each differentiated wavepacket is normalized to the peak intensity of the transmitted wavepacket through the silicon film on a quartz substrate (hollow triangles in Fig. 5a). We experimentally determine the time ${t}_{0}$ as the maximum intensity in Fig. 5a. By addressing their measured velocities, Fig. 5b shows, the experimental data $\bar{I}\left({v}_{T}\right)$ (red diamonds), which basically obey the predicted parabolic formula (solid curve). We notice that the solid curve in Fig. 5b is not a fitting one; rather, it is based on the experimentally determined parameter *A*, independently obtained from the measurement shown. For Sample 2, we observe a relatively large deviation, which might be caused mainly by the imperfect normal incidence of the wavepackets on the metagrating. Since the customization of arbitrarily shaped pulses is still an emerging research area, generating arbitrarily tilted pulses with specific transverse velocities remains challenging. Although a dispersion-controlled metalens is employed here to produce the tilted pulses, the current approach is still limited in generating pulses with arbitrary spatiotemporal dispersion. As a result, only limited experimental data based on three fabricated metalenses are presented in this work. Nevertheless, these results convincingly provide experimental verifications for the parabolic relationship between the normalized intensity and the transverse shifting velocity. Note that because the transverse shifted velocities are comparable to the speed of light, the parabolic relationship offered by the spatiotemporal differentiation suggests an indirect method to characterize the transverse movement of front-tilted photonic wavepackets.

## Discussion

In conclusion, we experimentally demonstrate spatiotemporal differentiation computation on single ultrafast optical wavepackets using a mirror-symmetry-breaking metagrating. We achieve first-order spatiotemporal differentiation with experimental resolutions of approximately 14 μm (in space) and 260 fs (in time). This ultrafast information processing enables real-time computing computation on spatiotemporal structured wavepackets, surpassing the current capabilities of the state-of-the-art electronic instruments. As a result, spatiotemporal computing opens new avenues in the emerging field of space-time optics^51–53^, with promising applications in optical communication and processing^54–56^, high-harmonic generation^57–59^, and optical tweezers^60,61^.

Specifically, we have demonstrated that the spatiotemporal differentiator is beneficial for characterizing the transverse shifting speed of front-tilted optical pulses. This spatiotemporal differentiation effect enables the detection of transverse motion at extremely high speeds, approaching the speed of light. It complements traditional techniques such as the Doppler effect for detecting longitudinal velocities and the angular Doppler effect using structured light for measuring lateral velocities^62,63^.

In comparison with conventional optical computing based on spatial differentiation^28–38^, our experimental results demonstrate that spatiotemporal differentiators provide fundamental building blocks for spatiotemporal optical computation, incorporating both spatial and temporal processing^64–66^. In the present work, we propose a metalens-based method to precisely modulate dispersion and generate spatiotemporally structured light. Recent advances have focused on encoding information in the spatiotemporal domain and on generating spatiotemporal holograms and images within a single ultrafast optical pulse using spatial light modulators (SLMs)^14,23–26^. With the development of various cutting-edge SLMs capable of gigahertz modulation rates and low power consumption^15,67,68^, spatiotemporal computing holds promise for enabling high-dimensional structured light as advanced information carriers, paving the way for ultrafast optical computing.

## Materials and methods

### Numerical simulation and characterizing the transfer function of the first-order spatiotemporal differentiator

We consider that the incident (transmitted) field is expressed as ${E}_{{in}({tran})}\left(x,t\right)={S}_{{in}({tran})}\left(x,t\right){e}^{-i{\omega }_{0}t}$ and decompose ${S}_{{in}({tran})}\left(x,t\right)$ into a weighted superposition of plane waves via a Fourier transform: ${S}_{{in}({tran})}\left(x,t\right)=\iint {\widetilde{S}}_{{in}({tran})}\left({k}_{x},\Omega \right)\exp \left(i{k}_{x}x-i\Omega t\right)d{k}_{x}d\Omega$, where ${S}_{{in}({tran})}$ and ${\widetilde{S}}_{{in}({tran})}$ denote the envelope amplitude and envelope spectrum of the incident (transmitted) field, respectively. ${k}_{x}$ is the transverse wavevector, and $\Omega ={\rm{\omega }}-{\omega }_{0}$ denotes the shifted temporal frequency with the center frequency ${\omega }_{0}$ of the wavepacket. One can evaluate the wavepacket transformation from the incident to the transmitted light by using the transfer function ${H\equiv \widetilde{S}}_{{tran}}\left({k}_{x},\Omega \right)/{\widetilde{S}}_{{in}}\left({k}_{x},\Omega \right)$ in momentum space.

Here, we numerically simulate the transfer function *H* via the finite-element method via the commercial full-wave software package COMSOL (SM Fig. S1), where the periodic boundary along the *x* direction is used. In our simulation, the refractive indices of silicon refer to the experimental data^69^. The design of the metagrating is implemented by scanning the structural parameters until the expected transfer function is obtained while considering the breaking of the mirror symmetry.

To characterize its transfer function, we develop homemade angle-resolved optical spectroscopy (see its optical setup in SM Fig. S2), which can output transmission at spatial and spectral frequencies. Owing to the broken mirror symmetry of our metagrating, the measured transfer function exhibits an asymmetric distribution of ${k}_{x}=0$, which can also be observed in the simulated transfer function (Fig. 1b). The measured transfer function at ${k}_{x}=0$ has a valley at a wavelength of ~1567 nm, which deviates slightly from the designed 1560 nm due to measurement uncertainty and fabrication errors. To evaluate the experimental ${C}_{x}$ and ${C}_{t}$, both the measured and simulated transfer functions are compared at the temporal and spatial frequencies (Fig. 1c), where their good agreement confirms the required linear dependence in a first-order spatiotemporal differentiator. In addition, its ability to generate spatiotemporal phase singularity has also been validated experimentally by checking the intensity and phase profiles of the wavepackets transmitted through this metagrating (see the simulated and experimental results in SM Fig. S3).

### Fabrication of metagrating and metalens

To fabricate the 3-dimensional metagrating with three-level heights, we use a double-exposure E-beam lithography process, which can be divided into three steps (SM Fig. S4). Step 1 is the generation of the mark for the next alignment. After the exposure and development of the photoresist (S1813), a 10 nm chromium film is deposited on the device via electron beam evaporation (Kurt J. Lesker, PVD75 Proline) to increase its adhesion, followed by the deposition of an 80 nm gold film. The cross-mark patterns are subsequently transferred into the Cr/Au film after lift-off in NMP (N-methylpyrrolidone) solvent. Step 2 is the generation of the first pattern layer. The positive electron-beam resist (AR-P 6200) is coated and baked. After alignment with cross-marks, the dried photoresist was patterned via electron beam lithography (JEOL, JBX 6300FS) and developed in AR600-546 for 60 s. The a-Si film without a photoresist is then etched to the designed thickness via an inductively coupled plasma-reactive ion etching (ICP-RIE) system (Oxford, Plasma Pro System100 ICP380). Finally, the residual photoresist is removed by the NMP solvent. Step 3 is the generation of the second pattern layer, which is a repetition of step 2 via electron-beam lithography but with different patterns. Then, the device is etched, and the photoresist is removed, yielding 3-dimensional metagrating.

Note that the feature size of the device is ~100 nm. To obtain high-quality metagrating, the first and second pattern layers must align precisely for better performance. To solve this problem, we fabricate a series of arrays of the first pattern layer with a fixed period of 500 μm. However, in the fabrication of the second pattern layer, the fixed period of the arrays is 499.99 μm, which is 10 nm smaller than that of the first pattern layer. This design works via the same principle with a Vernier calliper. Even if there are errors in alignment during fabrication, we can always find a relatively precise sample, which greatly reduces the difficulty of alignment issues in fabrication.

In this work, metalenses are also used to generate dispersion-engineered laser wavepackets (SM Sec. 1). Its fabrication follows the standard lift-off electron beam lithography process. First, a positive electron‒beam resist is patterned and developed, followed by deposition of a 10-nm-thick chromium film for hard masking. Thus, the nanobrick patterns are transferred into the ultrathin chromium film after lift-off. Then, the a-Si film without a hard mask is etched sufficiently by an ICP-RIE system. Finally, the residual chromium mask is removed by a chromium etchant, leaving the metalenses used in our experimental measurement.

## Supplementary information

Supplemental Material

## Data Availability

The data that support the findings of this study are available from the corresponding authors upon request.
