# Supplementary material for: Experimental demonstration of spatiotemporal analog computation in ultrafast optics
Source: Light Sci Appl. 2026 Jan 22;15:77. doi: 10.1038/s41377-025-02109-0 (PMC12824168; doi:10.1038/s41377-025-02109-0)
Supplement: Supplementary file 1 — Supplemental Material [file 41377_2025_2109_MOESM1_ESM.docx]

Supplementary Materials for

**Experimental demonstration of spatiotemporal analog computation in ultrafast optics**

Junyi Huang^1,#^, Dong Zhao^2,#^, Jixuan Shi^2,3,#^, Hongliang Zhang^1,#^, Hengyi Wang^2^,

Fang-Wen Sun^2,4^, Qiwen Zhan^5^, Shiyao Zhu^1,4,6^, Kun Huang^2,7,*^ , Zhichao Ruan^1,6**^

^1^ School of Physics, State Key Laboratory of Extreme Photonics and Instrumentation, and Zhejiang Province Key Laboratory of Quantum Technology and Device, Zhejiang University, Hangzhou 310027, China

^2^ Department of Optics and Optical Engineering, University of Science and Technology of China, Hefei 230026, China

^3^ Department of Physics, Tsinghua University, Beijing 10010, China

^4^ Hefei National Laboratory, Hefei, China.

^5^School of Optical-Electrical and Computer Engineering, University of Shanghai for Science and Technology, Shanghai 200093, China

^6^ College of Optical Science and Engineering, Zhejiang University, Hangzhou 310027, China

^7^State Key Laboratory of Opto-Electronic Information Acquisition and Protection Technology, School of Physical Sciences, University of Science and Technology of China, Hefei, Anhui 230026, China

# These authors contributed equally to this work

* huangk17@ustc.edu.cn

** zhichao@zju.edu.cn

### Section 1. Generating wavefront-tilted wavepackets via metalenses and experimental characterization

To realize tilted-front wavepackets, we employ propagation dispersion of the ultrafast wavepacket after passing through the metalenses with off-centered phase profiles of

$$\varphi\left( x,y \right)\text{=}exp\left[ ik_{0}\left( f\text{-}\sqrt{{(x\text{-}S_{x})}^{2}\text{+}y^{2}\text{+}f^{2}} \right) \right]$$

where the wavenumber $k_{0}\text{=}2\pi/\lambda_{0}$, $\lambda_{0}$ is the center wavelength of the incident light, $f$ is the focal length of the metalens, the eccentric distance $S_{x}$ is dependent on the radial position, the focal length $f\text{=}250$ μm is used in this work, and *x* and *y* are the Descartes’ coordinates. The eccentric phase makes our method fundamentally different from the frequency-graded phase. Owing to the propagation delay caused by different optical paths, the incident wavepacket at the radial position of the metalenses arrives at the focal plane in different sequential times so that the eccentric phase with *x*-coordinate dependence leads to a laterally moving focus with time, i.e., spatiotemporal front-tilted wavepackets. This proposal is validated theoretically by our numerical simulations with time-harmonic Rayleigh‒Sommerfeld diffraction. Three metalenses with different *S_x_* values are exemplified for the creation of front-tilted wavepackets (see their simulated spatiotemporal intensity profiles in Fig. S5).

The dispersion is caused by the optical path difference of light incident on the different radial positions of the metalenses. To tune the lateral shift of the focal spot via propagation-induced dispersion, the metalens has a modified phase profile in Eq. (4) of the main text, where the center of each radial phase shifts with the radial position. This means that the metalens has an eccentric phase, which is fundamentally different from the combination of tilting and a focusing phase. To maintain the intensity uniformity of the front-tilted wavepacket, one example of the eccentric distance is suggested with the form of $S_{x}\text{=}C\cdot\left[ tanh[d_{r}/D]\text{-}0.5 \right]$, where the dimensionless parameter $d_{r}\text{=}\left[ \sqrt{r^{2}+f^{2}}\text{-}f \right]/{\lambda_{0}}$ is the ratio of the optical path difference to the central wavelength, and the constants $C$ and $D$ are used to control the eccentric distances. Thus, we can use different $C$ and $D$ values to obtain various shifted speeds of the tilted wavepackets. For example, when $C\text{=}0$, we obtain a wavepacket with a shifted speed of zero (Fig. S5d), which is related to sample 1. When $C\text{=}3$ μm and $D\text{=}180$ for sample 2, a wavepacket with a shifted speed of $0.1507c_{0}$ (after being magnified by the experimental beam expander) is achieved, as shown in Fig. S5e. For sample 3, the wavepacket in Fig. S5f has a shifted speed of $0.2061c_{0}$ (after being magnified by the experimental beam expander), with parameters of $C\text{=}3.4$ μm and $D\text{=}200$. The corresponding phase profiles can be found in Figs. S5a-c.

The time-dependent focal fields of the metalenses under the different parameters are calculated via the time-harmonic Rayleigh‒Sommerfeld integral.

$E\left( x',y',z,t \right)\text{=}\frac{-1}{2\pi}\int\int\int E_{0}(x,y,\omega)\varphi\left( x,y,\omega\right)\frac{\partial}{\partial z}\left[ \frac{e^{ikR}}{R} \right]e^{-i\omega t}dxdyd\omega$ (S1)

where $E_{0}$ is the electric field of incident light, $k\text{=}\omega/c_{0}$, and $R^{2}\text{=}{(x\text{-}x')}^{2}\text{+}{(y\text{-}y')}^{2}\text{+}z^{2}$. To calculate the focal fields, the numerical integral of Eq. (S1) is used in the scientific computing software. The time-dependent intensity profiles of these three wavepackets generated directly by the proposed metalenses are provided in Figs. S5d-f.

To realize the designed phase, we use geometric metasurfaces that offer high-accuracy phase manipulation of circularly polarized light through the in-plane rotation of dielectric nanobricks. Experimentally, amorphous silicon nanoricks with less absorption at near-infrared wavelengths are employed here in one unit cell (see Fig. 2a in the main text) with a period of 450 nm along both the *x-* and *y*-directions. The geometric dimension of the nanobricks is designed via the finite-difference time-domain (FDTD) method, which can simulate the electromagnetic response and calculate the conversion efficiency from circular polarization to crossed polarization. The optimized silicon nanobricks with a period of 450 nm have a width of 150 nm, a length of 410 nm and a height of 760 nm.

### Section 2. Properties of the spatiotemporally differentiated front-tilted wavepackets.

To investigate the physical properties of the differentiated font-tilted wavepackets for a better experimental measurement, we give a brief derivation of the analytical formula for the differentiated wavepackets and reveal the dependence of the intensity on time. According to Eq. (2) in the main text, we have the intensity of the differentiated pulse

$$I_{v_{T}}\left( x,t \right)=\left| S_{tran}\left( x,t \right) \right|^{2}$$

$$=C_{x}^{2}e^{-\frac{2\left( t-t_{0} \right)^{2}}{w_{t}^{2}}}\cdot\left[ {\left( 1\text{+}Av_{T} \right)\cdot f}^{'}\left( x\text{-}v_{T}\left( t-t_{0} \right) \right)\text{+}\frac{2A\left( t-t_{0} \right)}{{w_{t}}^{2}}f\left( x\text{-}v_{T}\left( t-t_{0} \right) \right) \right]\cdot\left[ {\left( 1\text{+}A^{*}v_{T} \right)\cdot f}^{'}\left( x\text{-}v_{T}\left( t-t_{0} \right) \right)\text{+}\frac{2A^{*}\left( t-t_{0} \right)}{{w_{t}}^{2}}f\left( x\text{-}v_{T}\left( t-t_{0} \right) \right) \right]$$

$$=C_{x}^{2}e^{-\frac{2\left( t-t_{0} \right)^{2}}{w_{t}^{2}}}\left[ \left( 1\text{+}Av_{T} \right)\left( 1\text{+}A^{*}v_{T} \right){f^{'}}^{2}\text{+}\left( 2\text{+}Av_{T}\text{+}A^{*}v_{T} \right)\frac{2A\left( t-t_{0} \right)}{{w_{t}}^{2}}ff^{'}+\frac{4\left| A \right|^{2}\left( t-t_{0} \right)^{2}}{{w_{t}}^{4}}f^{2} \right]$$

$=C_{x}^{2}e^{-\frac{2\left( t-t_{0} \right)^{2}}{w_{t}^{2}}}g(x,t)$ (S2)

where the function *f* and its derivative $f^{'}$ are assumed to take the real values for the amplitude profiles of the pulses, and

$g\left( x,t \right)=\left[ \left( 1\text{+}Av_{T} \right)\left( 1\text{+}A^{*}v_{T} \right){f^{'}}^{2}\text{+}\left( 2\text{+}Av_{T}\text{+}A^{*}v_{T} \right)\frac{2A\left( t-t_{0} \right)}{{w_{t}}^{2}}ff^{'}+\frac{4\left| A \right|^{2}\left( t-t_{0} \right)^{2}}{{w_{t}}^{4}}f^{2} \right]$ (S3)

Thus, the time-dependent power of the differentiated pulse can be taken as

$P_{v_{T}}\left( t \right)=\int I\left( x,t \right)dx=C_{x}^{2}e^{-\frac{2\left( t-t_{0} \right)^{2}}{w_{t}^{2}}}\int g\left( x,t \right)dx$ (S4)

To reveal the time-dependent relationship, we can carry out the differentiation over Eq. (S4) with respect to time *t*, yielding

$\frac{\partial P_{v_{T}}\left( t \right)}{\partial t}=C_{x}^{2}e^{-\frac{2\left( t-t_{0} \right)^{2}}{w_{t}^{2}}}(-\frac{4\left( t-t_{0} \right)}{w_{t}^{2}})\int g\left( x,t \right)dx+C_{x}^{2}e^{-\frac{2\left( t-t_{0} \right)^{2}}{w_{t}^{2}}}\int\frac{\partial g\left( x,t \right)}{\partial t}dx$. (S5)

At the time *t*=*t_0_*, one can find that Eq. (S5) is zero after carrying out breif derivation. In Eq. (S5), the first item on the right-hand side is zero at *t*=*t_0_*. For the second item, we have

$$\int\frac{\partial g\left( x,t \right)}{\partial t}dx=\left( 1\text{+}Av_{T} \right)\left( 1\text{+}A^{*}v_{T} \right)\int\frac{\partial{f^{'}}^{2}}{\partial t}dx+\left( 2\text{+}Av_{T}\text{+}A^{*}v_{T} \right)\frac{2A}{{w_{t}}^{2}}\int\frac{\partial(\left( t-t_{0} \right)ff')}{\partial t}dx+\frac{4\left| A \right|^{2}}{{w_{t}}^{4}}\int\frac{\partial\left( \left( t-t_{0} \right)^{2}f^{2} \right)}{\partial t}dx$$

$=-2\left( 1\text{+}Av_{T} \right)\left( 1\text{+}A^{*}v_{T} \right)v_{T}\int f^{'}f^{''}dx+\left( 2\text{+}Av_{T}\text{+}A^{*}v_{T} \right)\frac{2A}{{w_{t}}^{2}}\left[ \int ff'dx+\left( t-t_{0} \right)\int\frac{\partial(ff')}{\partial t}dx \right]+\frac{4\left| A \right|^{2}}{{w_{t}}^{4}}\left[ 2\left( t-t_{0} \right)\int f^{2}dx+\left( t-t_{0} \right)^{2}\int\frac{\partial\left( f^{2} \right)}{\partial t}dx \right]$ (S6)

In Eq. (S6), when *t*=*t_0_*, $\int_{-\infty}^{\infty} f'f^{''}dx=\frac{1}{2}{f'}^{2}|_{-\infty}^{\infty}=\frac{1}{2}{f'\left( \infty\right)}^{2}-\frac{1}{2}{f^{'}\left( -\infty\right)}^{2}=0$ and $\int_{-\infty}^{\infty} ff^{'}dx=\frac{1}{2}{f\left( \infty\right)}^{2}-\frac{1}{2}{f\left( -\infty\right)}^{2}=0$ because $f^{'}\left( \pm\infty\right)=f\left( \pm\infty\right)=0$ for the finite size of a realistic beam of light. After rechecking Eq. (S6), one can find that $\int\frac{\partial g\left( x,t \right)}{\partial t}dx\text{=}0$ at *t*=*t_0_*.

${\partial P_{v_{T}}\left( t \right)}/{\partial t}\text{=}0$ indicates that the maximum or minimum power of the differentiated pulse occurs at *t*=*t_0_*, which helps the experimental implementation find the key time. For example, in this work, the function *f* has a Gaussian distribution so that it has a maximum power at *t*=*t_0_*, as observed in Fig. 5a in the main text. Importantly, the conclusion that the first derivative of the power $P_{v_{T}}$ is zero at *t*=*t_0_* is valid for any real function *f* of a realistic beam.


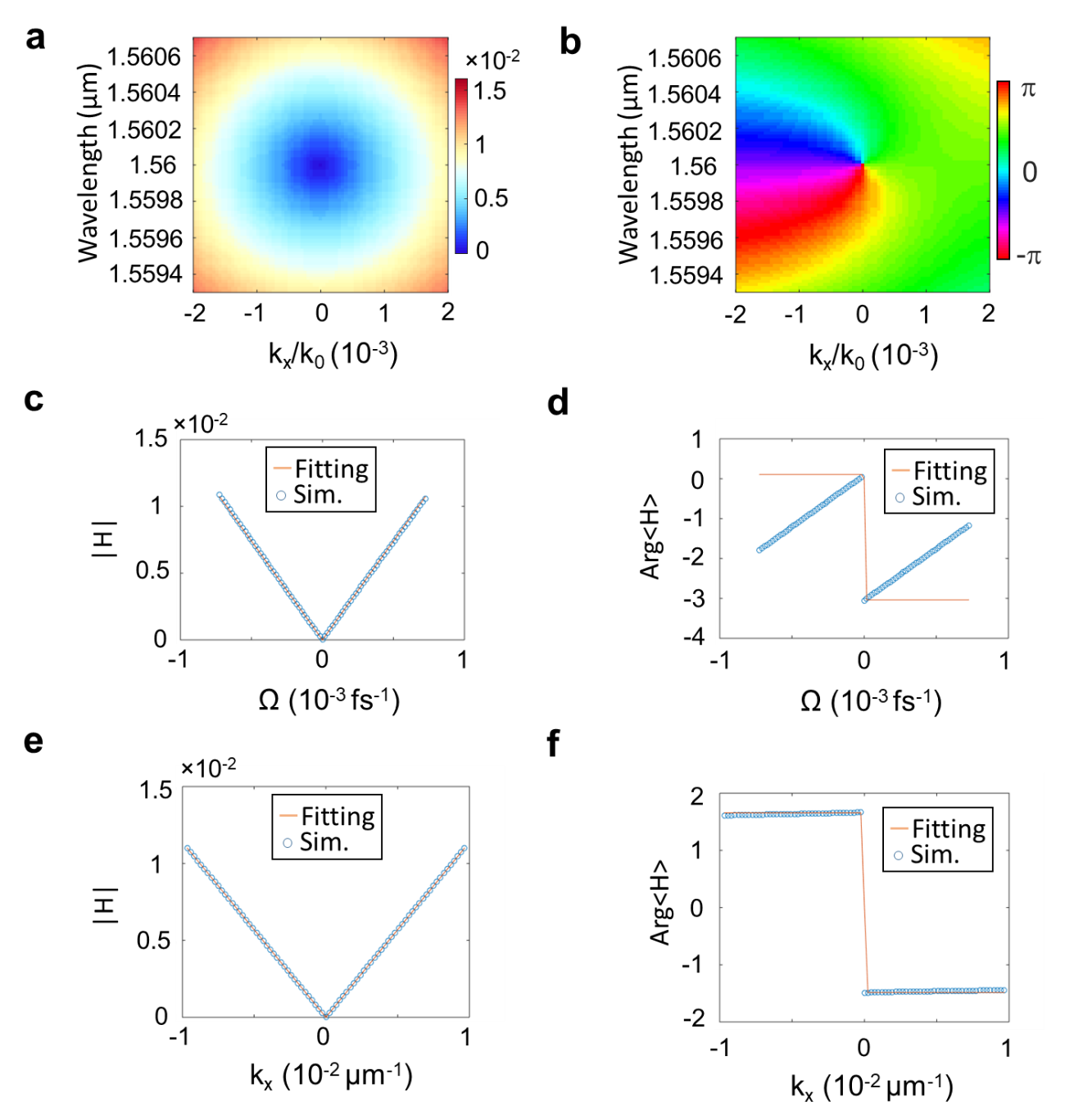


Figure S1. Simulated properties of the proposed spatiotemporal differentiators. **(a-b)** Simulated amplitude **(a)** and phase **(b)** of the transmission function H. **(c-d)** Line-scanning amplitude **(c)** and phase **(d)** of transmission function H with a variable temporal frequency Ω at *k_x_*=0. Linear fitting (solid lines) is employed to predict the differentiation strength *C_t_*. (e-f) Line-scanning amplitude **(e)** and phase **(f)** of transmission function H with a variable spatial frequency *k_x_* at Ω=0. Linear fitting (solid lines) is employed to predict the differentiation strength *C_x_*.


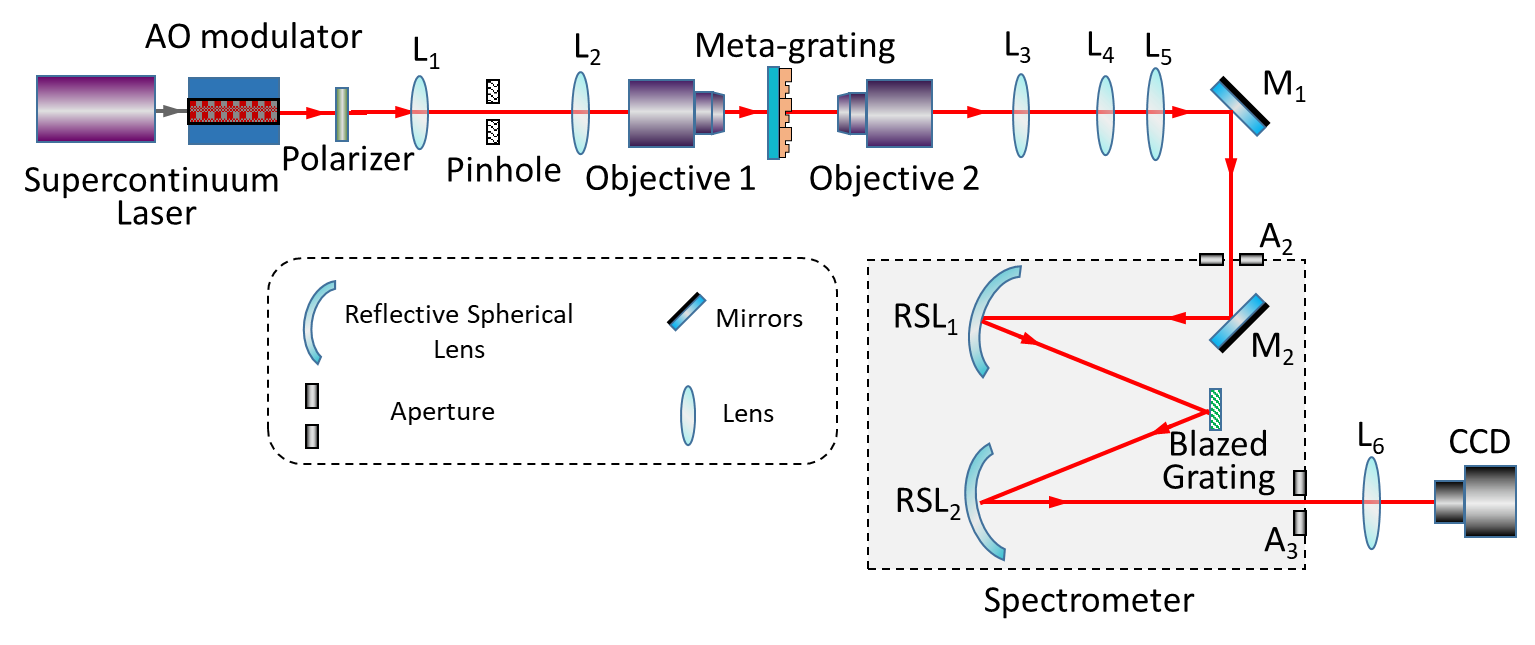


Figure S2. Setup for measuring the optical transmission |H|^2^ of our meta-grating. A supercontinuum laser (SuperK Fianium FIU-15) with a broadband operating spectrum from 410 nm to 2400 nm is filtered by an acoustic-optic (AO) modulator, yielding a quasimonochromic light source. To obtain the required polarization, the light passes through a linear polarizer before being expanded by a telescope system (composed of two lenses L_1_ and L_2_) and filtered spatially by a pinhole. To realize a large range of spatial frequencies, objective 1 is used to focus the light on our fabricated metagrating sample. After the transmitted light is collected by another objective lens 2, several lenses (L_3_, L_4_ and L_5_) are used to project the spatial frequency of our meta-grating onto entrance A_2_ of an optical spectroscope (composed of the key components: a blazed grating and two reflective spherical mirrors). In this spectroscopy, the conjugate port of A_2_ is exit A_3_, where the information of both spatial and temporal frequencies is located. To capture them, an additional imaging lens L_6_ is used to project the frequency information onto a CCD camera.


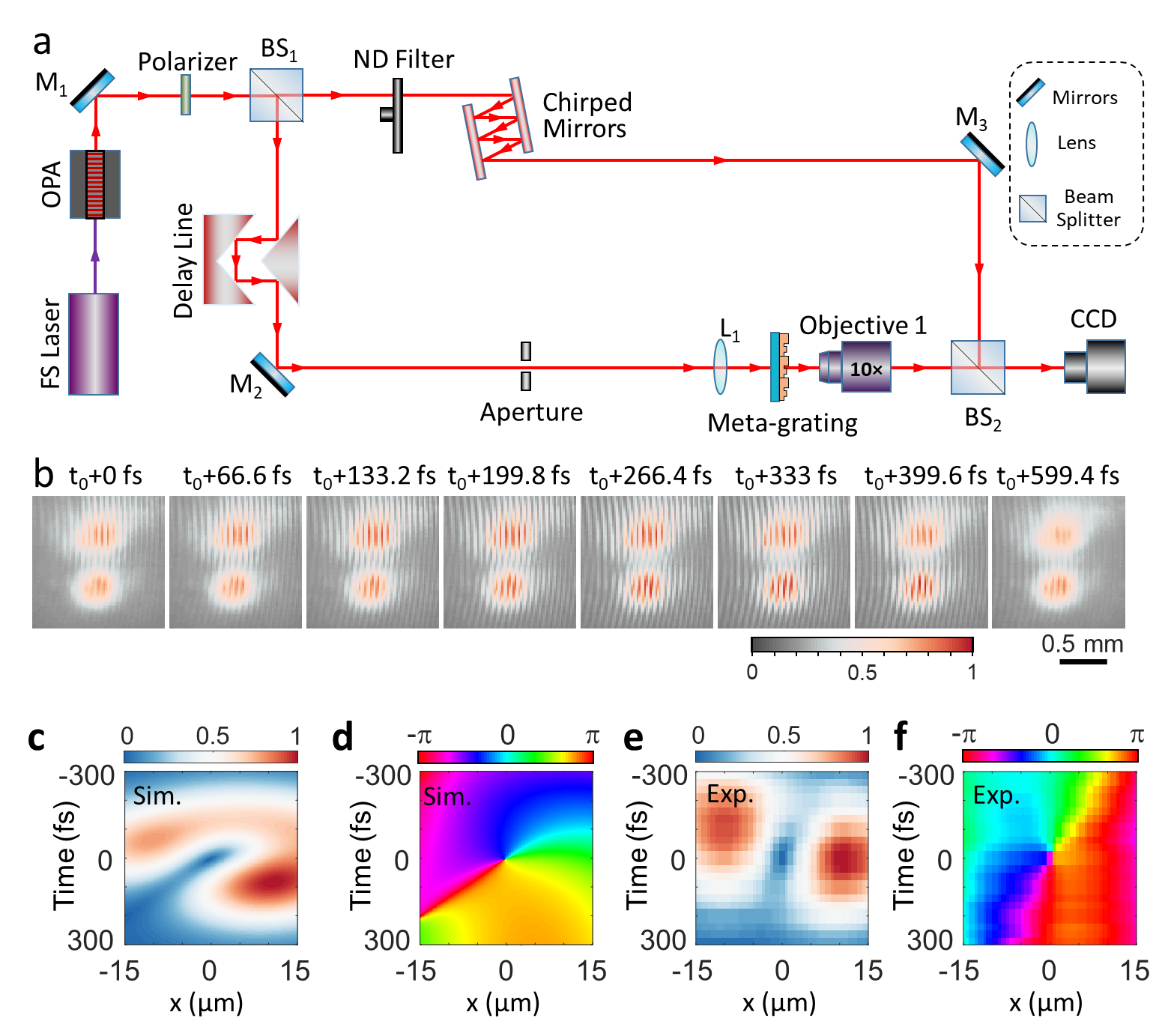


Figure S3. Characterization of our meta-grating-based spatiotemporal differentiator. **(a)** Optical setup for measuring the spatiotemporal properties of the pulse transmitted through our meta-grating in a Mach–Zender interferometer. In this interferometer, the pulse in the reference arm is suppressed by two chirped mirrors, whereas the delay line composed of two complementary prisms is located in the signal beam to tune the optical path for generation of the interference patterns, which are recorded by a CCD camera after being projected and magnified by objective 1. **(b)** Recorded interference patterns with a time interval of 33.3 fs. Dislocation between the upper and lower interference fringes occurs, implying the creation of spatiotemporal phase singularity. **(c-d)** Simulated intensity **(c)** and phase **(d)** profiles of a spatiotemporal pulse that passes through our meta-grating. The incident pulse has a Gaussian distribution with a central wavelength of *λ*_0_=1560 nm (with a spectral waist of 16 nm) and a Gaussian distribution of spatial frequencies with a spatial waist of 0.035*k*_0_ (where *k*_0_=2π/*λ*_0_). **(e-f)** Measured intensity **(e)** and phase **(f)** profiles of the pulse transmitted through the metagrating. Both the intensity and phase profiles are retrieved from the experimentally captured interference fringes. In the phase profiles, the temporal part is not considered.


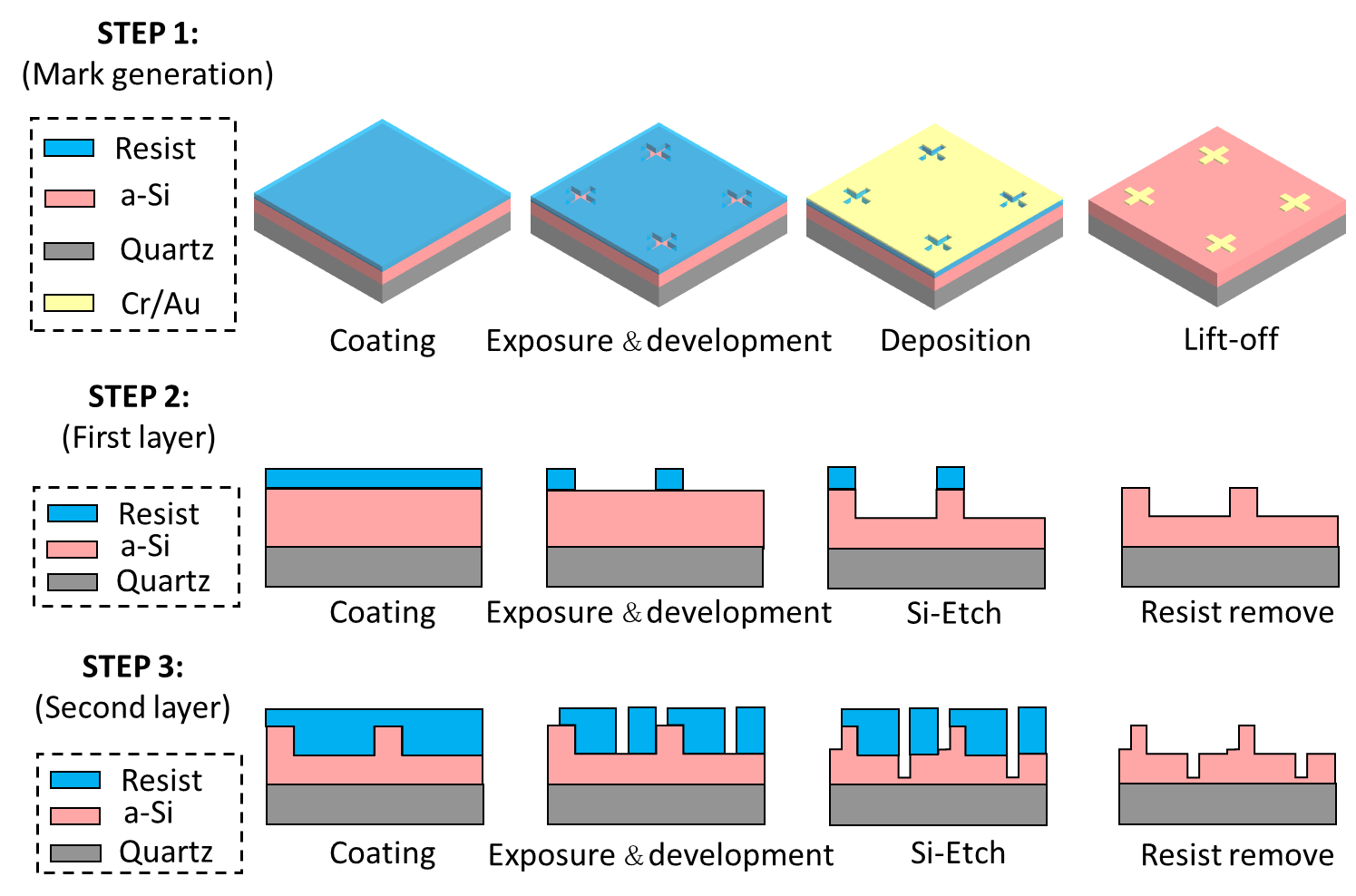


Figure S4. Fabrication sketch for our meta-grating-based spatiotemporal differentiators. The fabrication includes three processes: mark generation, first-layer patterning, and second-layer patterning. The details of the fabrication process are provided in the Methods section of the main text.


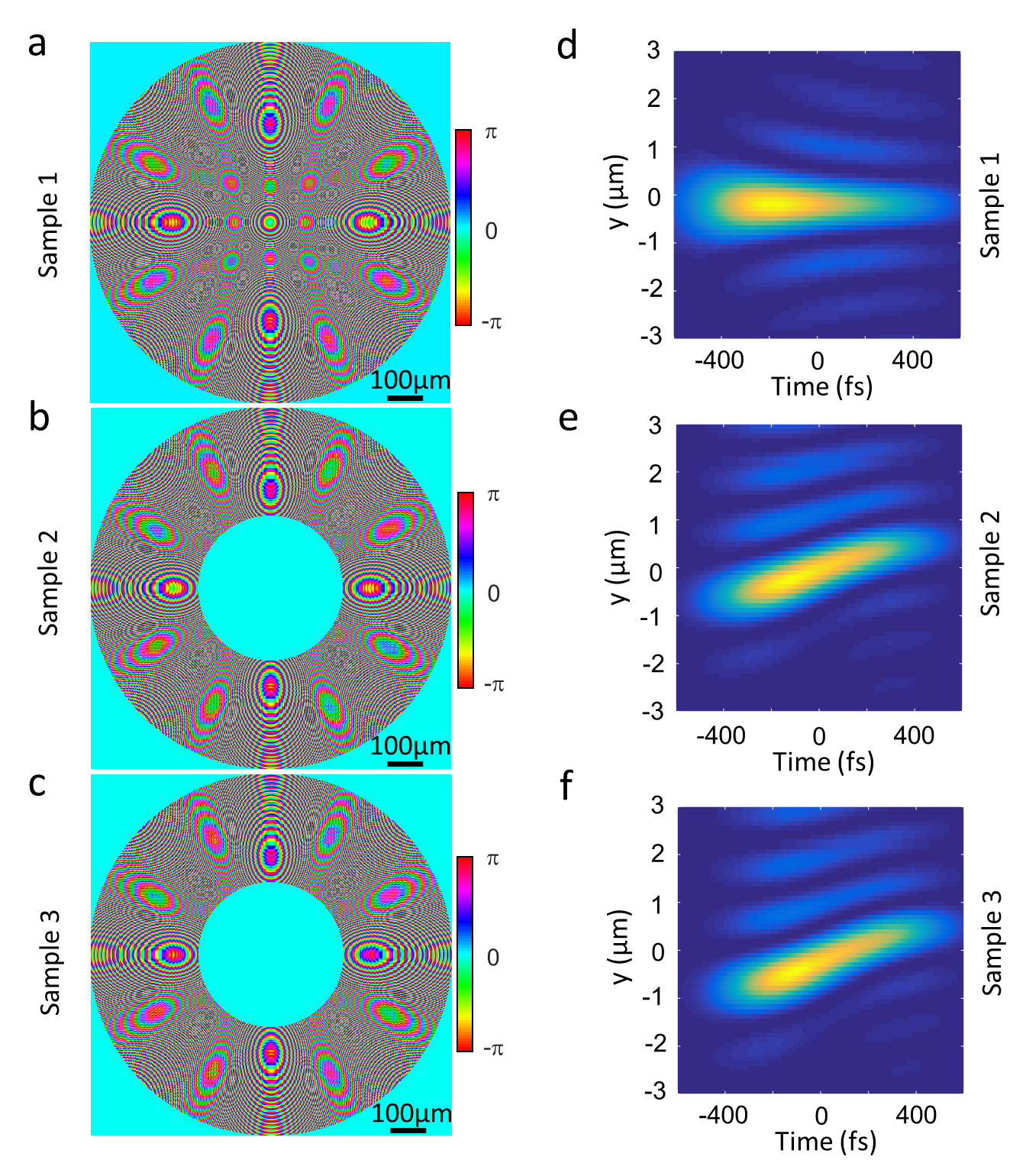


Figure S5. Optical properties of the dispersion-controlled metalenses**. (a-c)** Phase profiles encoded in three metalenses (samples 1, 2 and 3) used in this work. The central part is blocked to avoid the low transverse velocity in the generated front-titled wavepackets, which can be observed in the following simulated intensity profiles in (**d-f**). Note that such a blocking operation has no significant influence on the shape of the generated pulses, thereby leading to negligible effects on the experimental results because only the intensity at *t*=0 is used to demonstrate the proposed effect. **(d-f)** Time-dependent intensity profiles at the focal plane of the metalenses for samples 1 **(d)**, 2 **(e)** and 3 **(f)**.


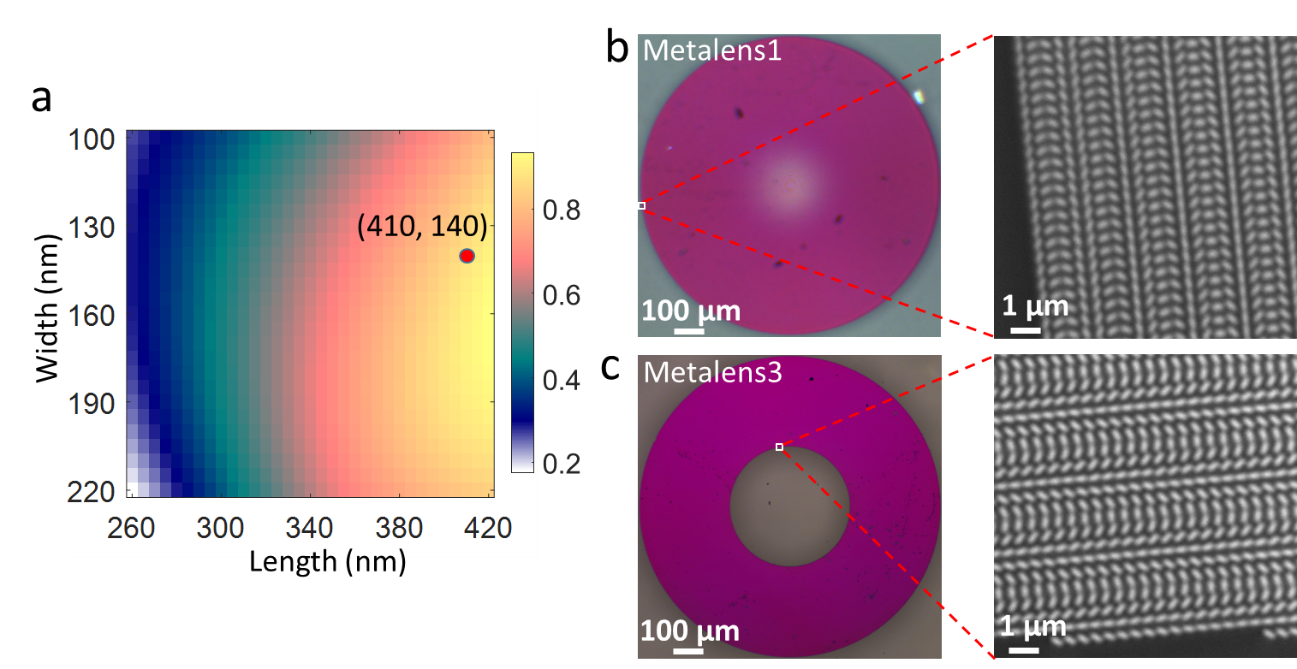


Figure S6. Dispersion-controlled metalenses. **(a)** Simulated efficiency of polarization conversion as a function of nanobrick geometry (*i.e.*, width and length). In this work, the 140-nm width and 410-nm length geometry of the nanobrick is used after the balance between efficiency and fabrication difficulty is reached. **(c)** Microscopy images of these three metalenses. The images in the left panel were taken with a 20X objective, whereas those in the right panel were taken with a 150X objective lens for better imaging resolution.


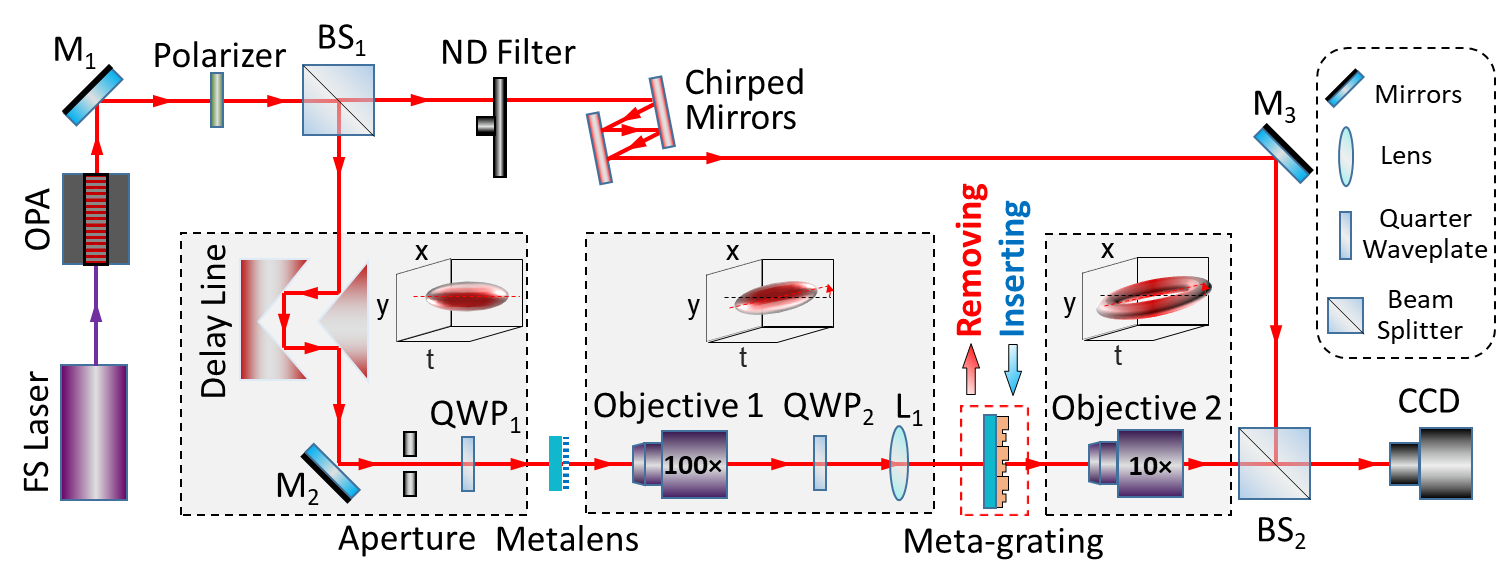


**Figure S7 Experimental setup for characterizing the creation of front-tilted wavepackets with a custom-made Mach–Zender interferometer**. The signal arm is responsible for the creation of front-tilted wavepackets. The signal arm contains three key parts: a delay line (the left dashed box) composed of two complementary V-shape prisms to measure the time-dependent interference fringes, a generator (the middle dashed box) of the tilted pulse by using the dispersion-controlled metalens, a meta-grating based differentiator (the right dashed box). In the middle box, an objective lens with its NA=0.9 is used to collect the tilted pulse generated by the metalens. The quarter waveplate (QWP_2_) can convert the circularly polarized light into the linearly polarized light, which is then taken as the incidence of the following meta-grating. To match the dimension of the incident spot, a spherical lens (L_1_) is employed to decrease the size of the incident linear-polarization light. In the right box, an objective lens with its magnification of 10 is used to project the light at the surface of the meta-grating onto the CCD, which can record the interference patterns. In the reference arm, two chirped mirrors are used to compensate the dispersion for obtaining a narrow reference pulse so that the experimental temporal resolution can be sufficient to resolve the time details of the processed pulses. In addition, it is important to note that, when the meta-grating is removed, the remaining system can be used to measure the spatiotemporal properties of the tilted pulse.


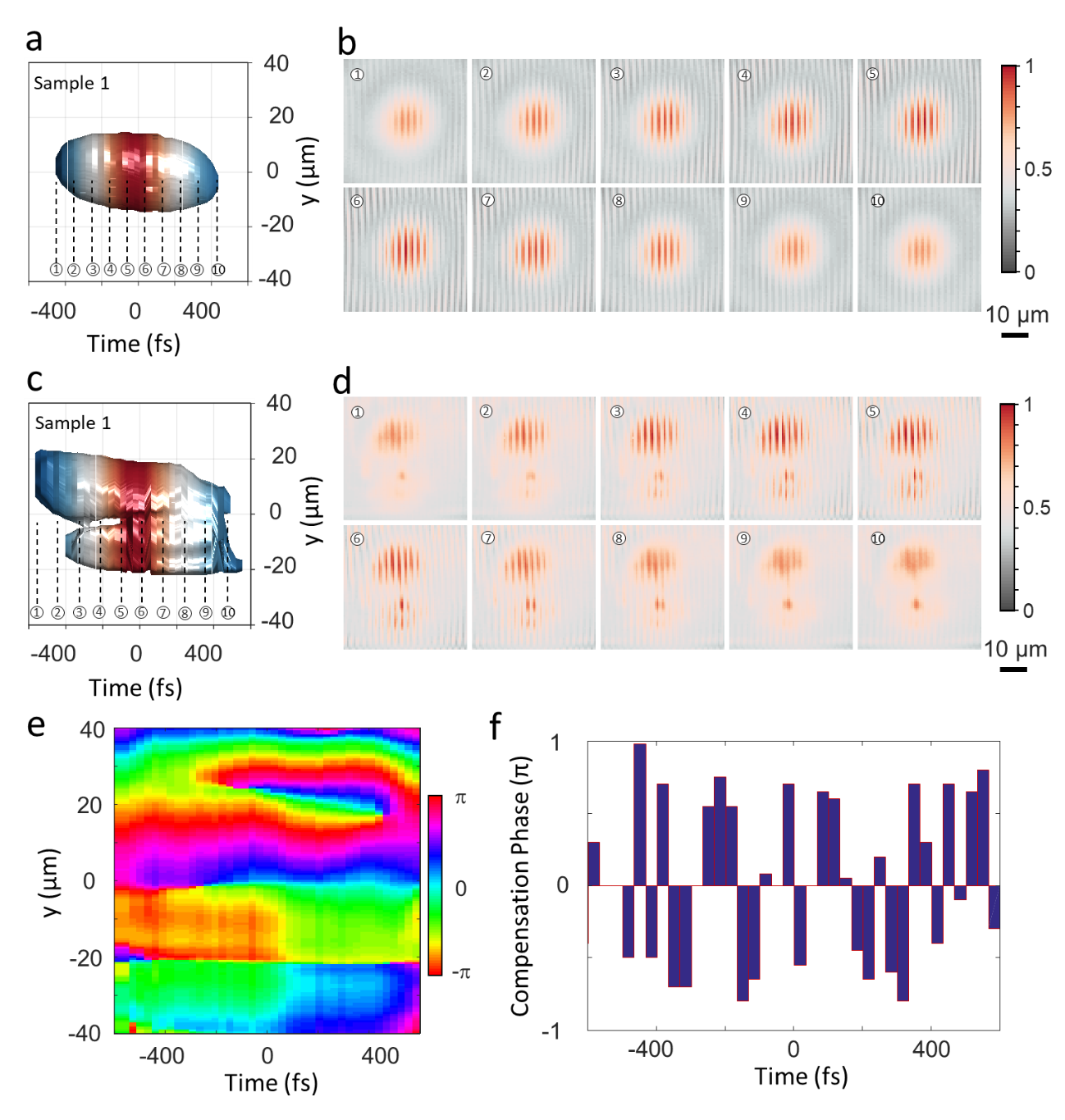


Figure S8. Experimental results for the incident pulse generated by metalens 1 (sample 1) and its differentiated pulse**. (a)** Retrieved iso-intensity profiles of incident pulses generated by sample 1. This is the same as in Fig. 2c in the main text. **(b)** Measured interference patterns with the temporal indices labelled in **(a)**. **(c)** Retrieved iso-intensity profiles of differentiated pulses via our meta-grating. This is the same as in Fig. 3b in the main text. **(d)** Measured interference patterns with the temporal indices labelled in **(c)**.


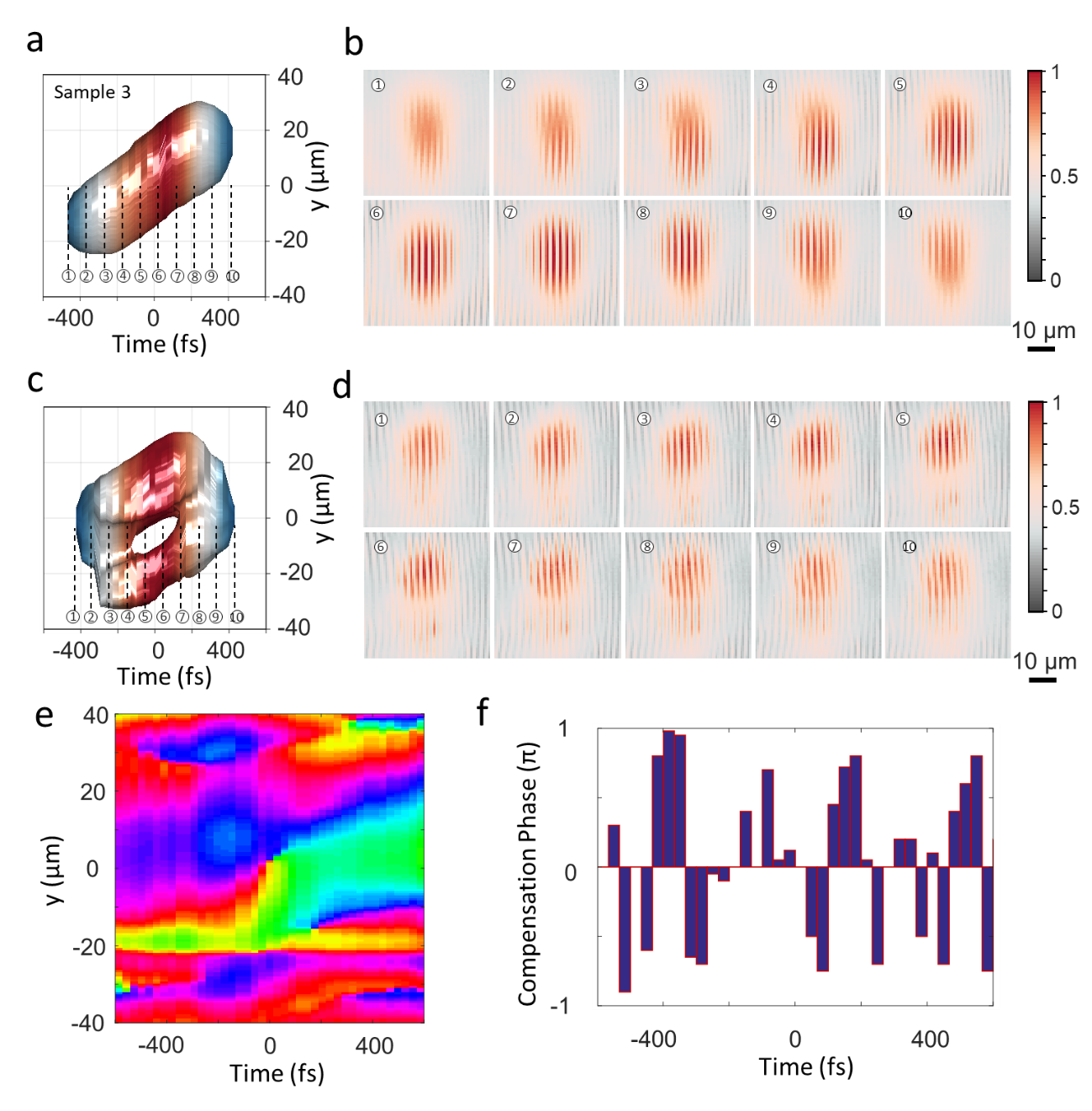


Figure S9. Experimental results for the incident pulse generated by metalens 3 (sample 3) and its differentiated pulse**. (a)** Retrieved iso-intensity profiles of incident pulses generated by sample 3. This is the same as in Fig. 2e in the main text. **(b)** Measured interference patterns with the temporal indices labelled in **(a)**. **(c)** Retrieved iso-intensity profiles of differentiated pulses via our meta-grating. This is the same as in Fig. 3d in the main text. **(d)** Measured interference patterns with the temporal indices labelled in **(c)**.
